# Supplementary material for: Temperature impacts the bovine ex vivo immune response towards Mycoplasmopsis bovis
Source: Vet Res. 2024 Feb 13;55:18. doi: 10.1186/s13567-024-01272-3 (PMC10863263; doi:10.1186/s13567-024-01272-3)
Supplement: Supplementary file 1 — Additional file 1: Characteristics of animals. [file 13567_2024_1272_MOESM1_ESM.pdf]

Animal	Location	Breed	ID	Age (years)	Sex
●	Agroscope	*Bos taurus*	CH120150276437	2	Female
■	Agroscope	*Bos taurus*	CH120150276420	2	Female
▲	Agroscope	*Bos taurus*	CH120150276581	1	Female
◆	Agroscope	*Bos taurus*	CH120150276451	2	Female
●	Agroscope	*Bos taurus*	CH120150276444	2	Female
▼	Agroscope	*Bos taurus*	CH120150276499	1	Female
●	Agroscope	*Bos taurus*	CH120150276482	1	Female
■	Agroscope	*Bos taurus*	CH120150276475	1	Female
△	Agroscope	*Bos taurus*	CH120150276550	1	Female
▽	Agroscope	*Bos taurus*	CH120150276567	1	Female
△	Agroscope	*Bos taurus*	CH120150276512	1	Female
▽	Agroscope	*Bos taurus*	CH120158564574	1	Female
○	Agroscope	*Bos taurus*	CH120150276574	1	Female
□	Agroscope	*Bos taurus*	CH120150276635	1	Female
○	Agroscope	*Bos taurus*	CH120150276628	1	Female
□	Agroscope	*Bos taurus*	CH120158564628	1	Female
●	Agroscope	*Bos taurus*	CH120150276505	2	Female
■	Agroscope	*Bos taurus*	CH120150276611	2	Female
▼	Agroscope	*Bos taurus*	CH120150276543	2	Female
▲	Agroscope	*Bos taurus*	CH120150276529	2	Female
△	Agroscope	*Bos taurus*	CH120150276581	1	Female
▽	Agroscope	*Bos taurus*	CH120150276468	2	Female
