# Supplementary material for: Temperature impacts the bovine ex vivo immune response towards Mycoplasmopsis bovis
Source: Vet Res. 2024 Feb 13;55:18. doi: 10.1186/s13567-024-01272-3 (PMC10863263; doi:10.1186/s13567-024-01272-3)
Supplement: Supplementary file 2 — Additional file 2: Antibodies against M. bovis were not detected in the serum of animals enrolled in the study. [file 13567_2024_1272_MOESM2_ESM.pdf]

A

Raw data

	1	2	3	4	5	6	7	8
**A**	0.066	0.065	0.104	0.057	0.082	0.057	0.07	0.071
**B**	0.039	0.092	0.09	0.059	0.086	0.06	0.073	0.073
**C**	1.276	0.066	0.079	0.085	0.063	0.29	0.092	0.071
**D**	1.154	0.063	0.1198	0.092	0.049	0.078	0.099	0.078
**E**	0.082	0.111	0.158	0.082	0.228	0.084	0.114	0.085
**F**	0.057	0.085	0.12	0.108	0.073	0.079	0.079	0.069
**G**	0.047	0.066	0.049	0.055	0.059	0.091	0.093	
**H**	0.055	0.083	0.046	0.043	0.058	0.076	0.07	

B

Analysed data

	1	2	3	4	5	6	7	8
**A**								
**B**	**0.0**	2.2	3.8	0.5	2.7	0.5	1.6	1.7
**C**								
**D**	**100.0**	1.0	4.0	3.1	0.3	11.3	3.7	1.9
**E**								
**F**	1.5	3.9	7.4	3.7	8.4	2.5	3.8	2.1
**G**								
**H**	-0.1	1.9	-0.4	-0.3	0.5	2.7	2.5	
