# Supplementary figures and images for: Temperature impacts the bovine ex vivo immune response towards Mycoplasmopsis bovis
Source: Vet Res. 2024 Feb 13;55:18. doi: 10.1186/s13567-024-01272-3 (PMC10863263; doi:10.1186/s13567-024-01272-3)

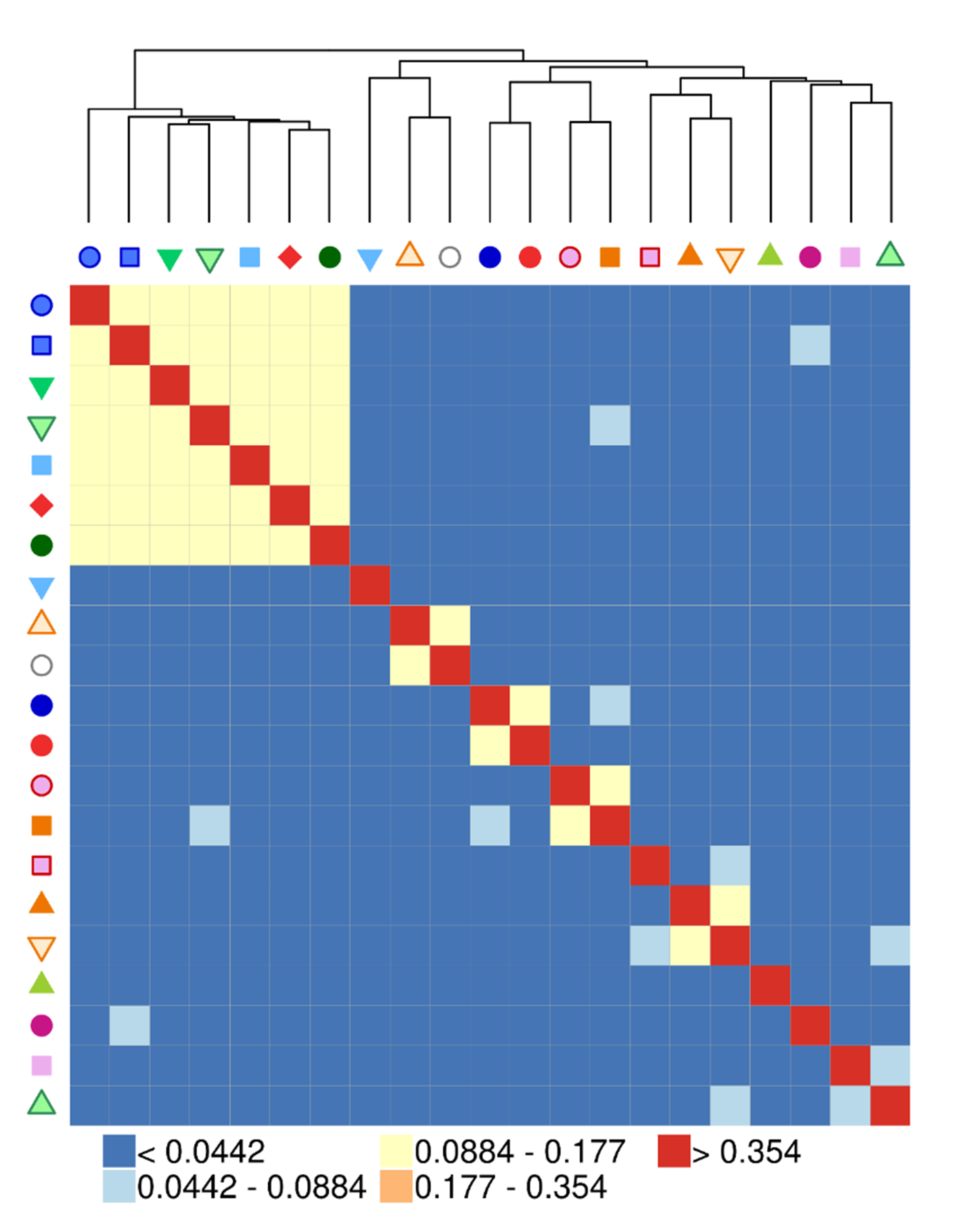

Supplement: Supplementary file 3 — Additional file 3: Heatmap of pairwise kinship estimates from genomic data. The dendrogram depicts clusters identified by the complete linkage method of the hclust function in R. Colors indicate relatedness according to KING cutoff values as duplicates/monozygotic twins (> 0.354; red), 1st-degree (0.177–0.354; orange), 2nd-degree (0.0884–0.177; yellow), or 3rd-degree relatives (0.0442–0.0884; light blue), and more distantly related/unrelated individuals (< 0.0442; blue). [file 13567_2024_1272_MOESM3_ESM.tif]

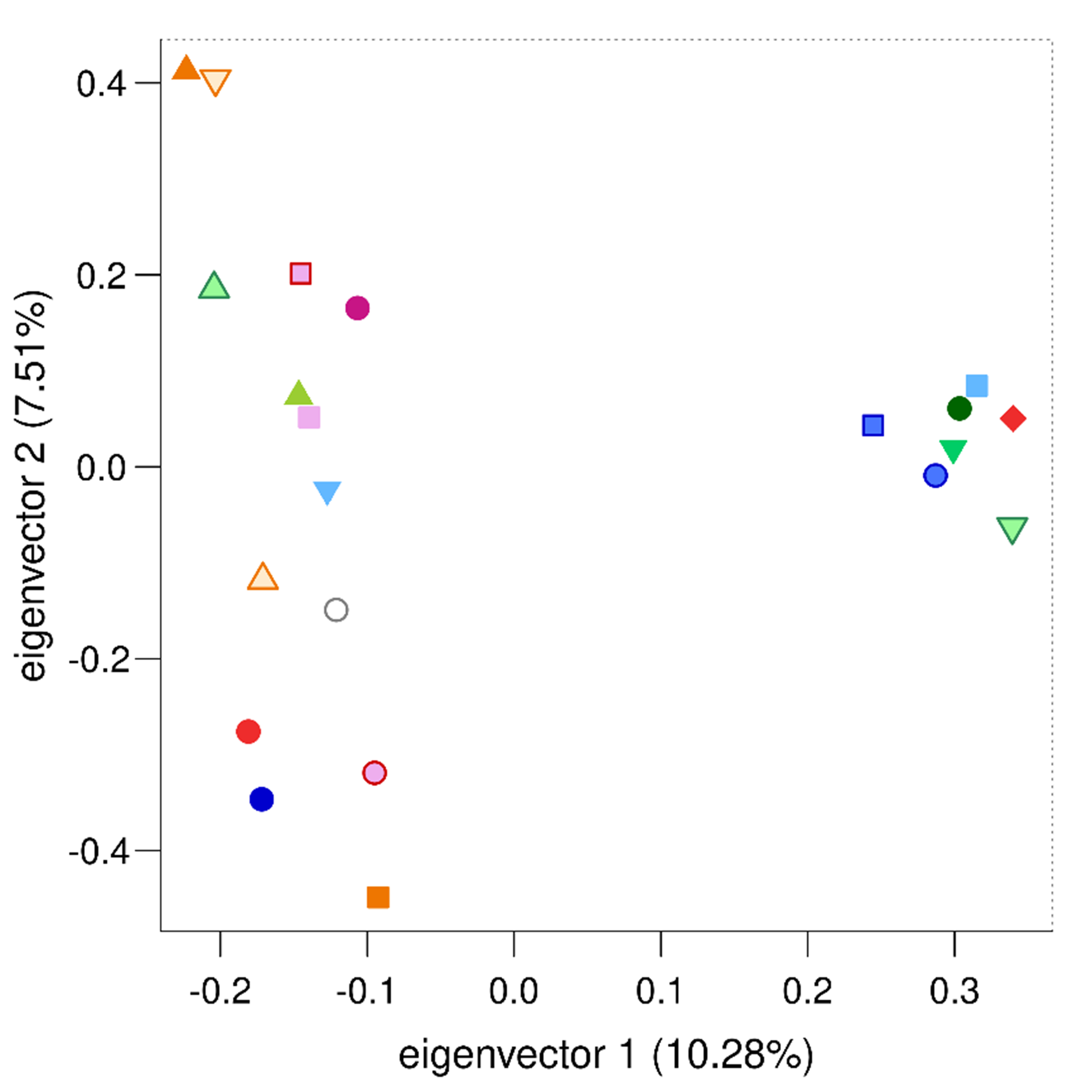

Supplement: Supplementary file 4 — Additional file 4: First and second principal components (eigenvector 1 and 2) estimated from genomic data by principal component analysis (PCA). Symbols represent individual cows. [file 13567_2024_1272_MOESM4_ESM.tif]

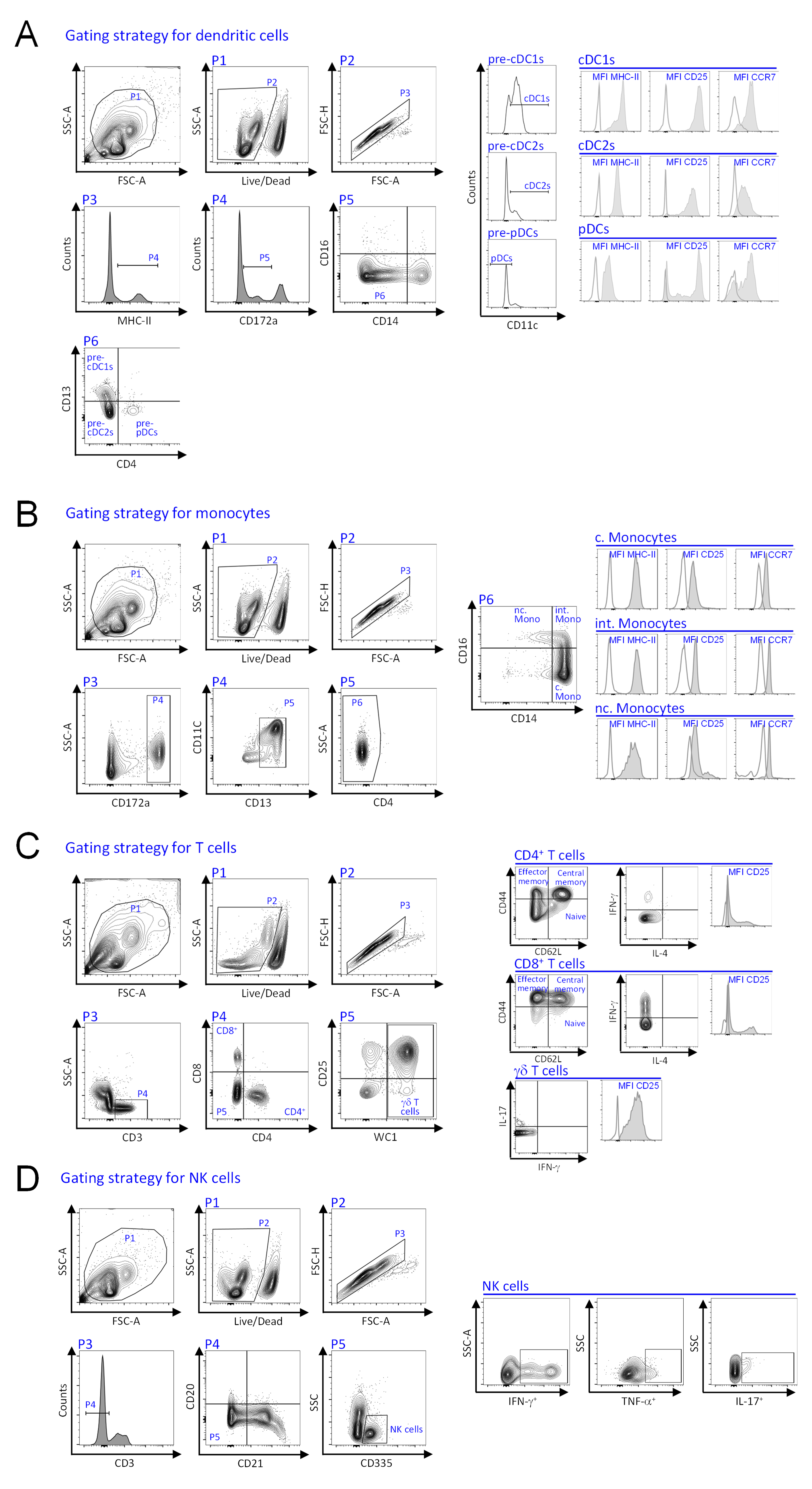

Supplement: Supplementary file 5 — Additional file 5: FCM gating strategy for immune cell identification. A Example of gating strategy for multiparameter FCM analysis of bovine DCs. Gating strategy following multiparameter FCM staining uses Abs against MHC-II, CD172a, CD16, CD14, CD13, CD4, CD11c, CD25 and CCR7. A primary gate (P1) was set on FSC-A versus SSC-A. Then, the dead cell population (positive for Live/Dead) was excluded, followed by a FSC-H/FSC-A contour plot (to exclude doublets from the analysis). Among these cells, we defined the population positive for MHC-II and CD172a (low and intermediate only). Then, we defined among this population the cells negative for both CD14 and CD16 markers. Then, we defined among this population either the cells positive for CD13 and negative for CD4 markers (pre-cDC1s), either cells negative for CD13 and positive for CD4 markers (pre-pDCs), either cells negative for both (pre-cDC2s). Next, we considered CD11c marker to gate cDC1s and cDC2s (respectively pre-cDC1s and pre-cDC2s positive CD11c), as well as pDCs (pre-pDCs negative for CD11c). Maturation was evaluated based on the MFI of surface expression for MHC-II, CD25 and CCR7. (B) Example of gating strategy for multiparameter FCM analysis of bovine monocytes. Gating strategy following multiparameter FCM staining uses Abs against MHC-II, CD172a, CD16, CD14, CD13, CD4, CD11c, CD25 and CCR7. A primary gate (P1) was set on FSC-A versus SSC-A. Then, the dead cell population (positive for Live/Dead) was excluded, followed by a FSC-H/FSC-A contour plot (to exclude doublets from the analysis). Next, we defined among this population the cells highly positive for CD172a marker (CD172ahigh cells), positive for CD13, and then negative for CD4. From this, classical monocytes (c. Mono) were gated as CD14+CD16-, intermediate monocytes (int. Mono) as CD14+CD16+, and nonclassical monocytes (nc. Mono) as CD14-CD16+. As for the DC substets, maturation was evaluated based on MFI of surface expression for MHC-II, CD25 and CC [file 13567_2024_1272_MOESM5_ESM.tif]

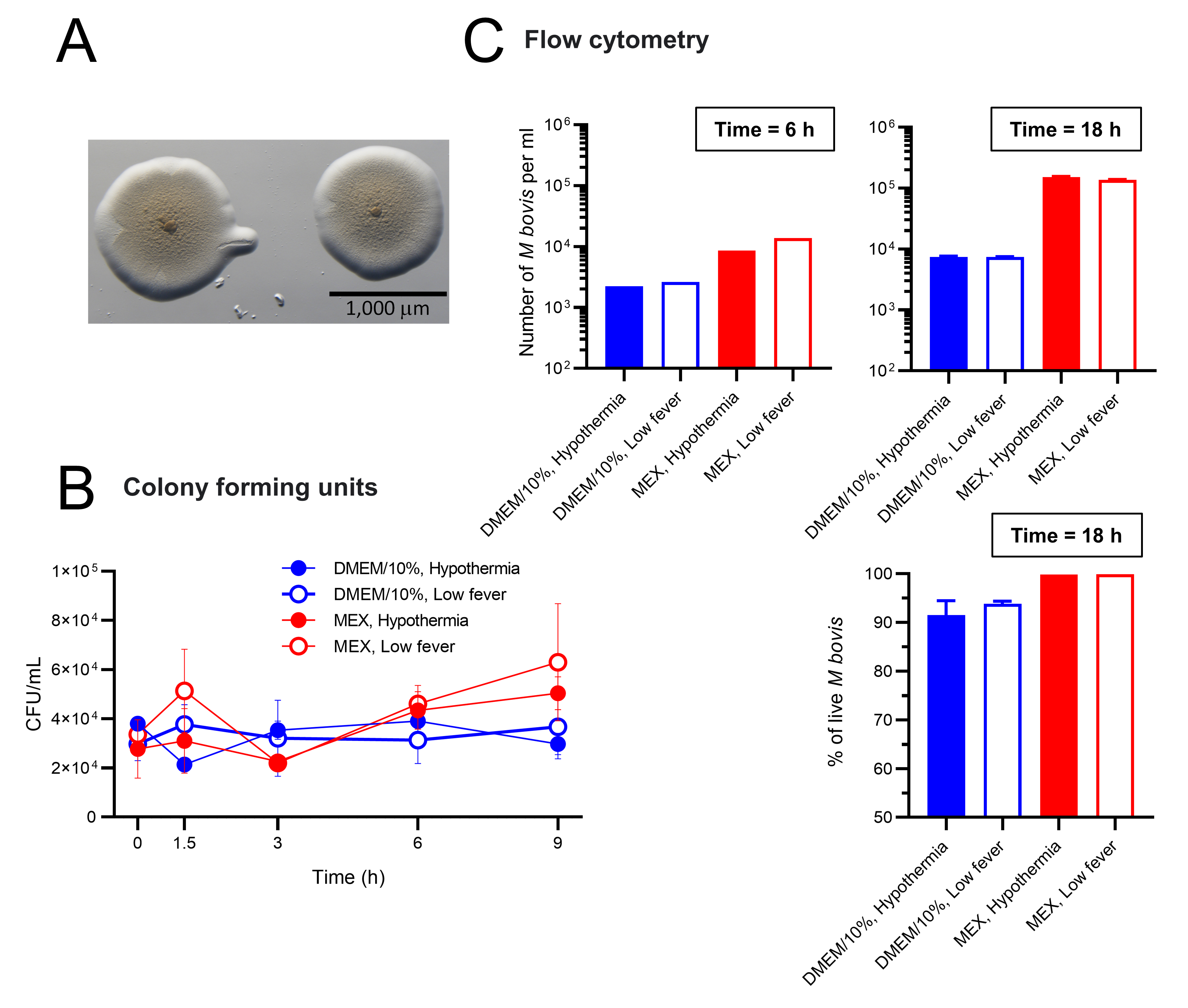

Supplement: Supplementary file 6 — Additional file 6: Mycoplasmopsis bovis survival in primary blood cell culture medium. A Colonies with typical M. bovis morphology (Donetta PG45 strain, used in the present study). The size bar displays 1000 μm. B CFU counts taken at different time points. M. bovis was grown in MEX or DMEM/10%, either at 37 °C (Hypothermia) or 39.5 °C (Low fever). C Similar as (B), but with FCM as readout. To determine the percentage of live M. bovis, BacLightTM RedoxSensorTM Green Vitality Kit was employed. [file 13567_2024_1272_MOESM6_ESM.tif]

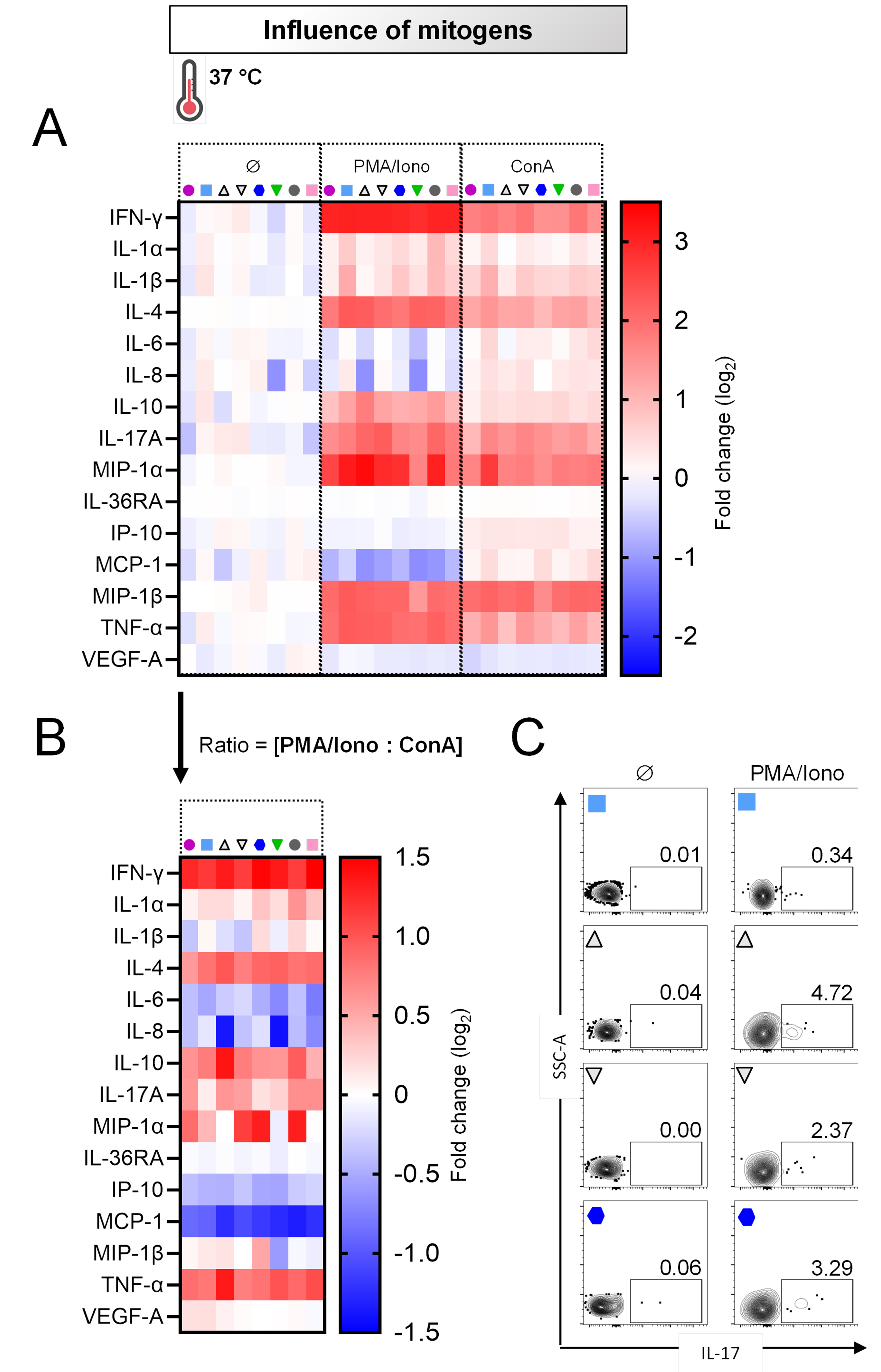

Supplement: Supplementary file 7 — Additional file 7: Induction of cytokines by primary blood cells following exposure to mitogens. (A) Cytokine / chemokine secretion in supernatants of PBMC cultures, using Multiplex immunoassay. Stimulations were run at 37 °C. A single measurement was done per samples tested, and each symbol represents an individual cow. Heat map shows log2-fold changes in concentration of 15 cytokines/chemokines. For a given cytokine / chemokine, normalization was as follow: [concentration for a given animal] / [average concentration of reference points (∅) for 8 animals]. (B) As in (A), but displaying the ratios: [PMA/Iono: ConA]. C Here are shown IL-17 producing cells among the γδ T cell fraction (FCM contour plots) for 4 animals. Numbers indicate the percentage of IL-17+ cells. [file 13567_2024_1272_MOESM7_ESM.tif]

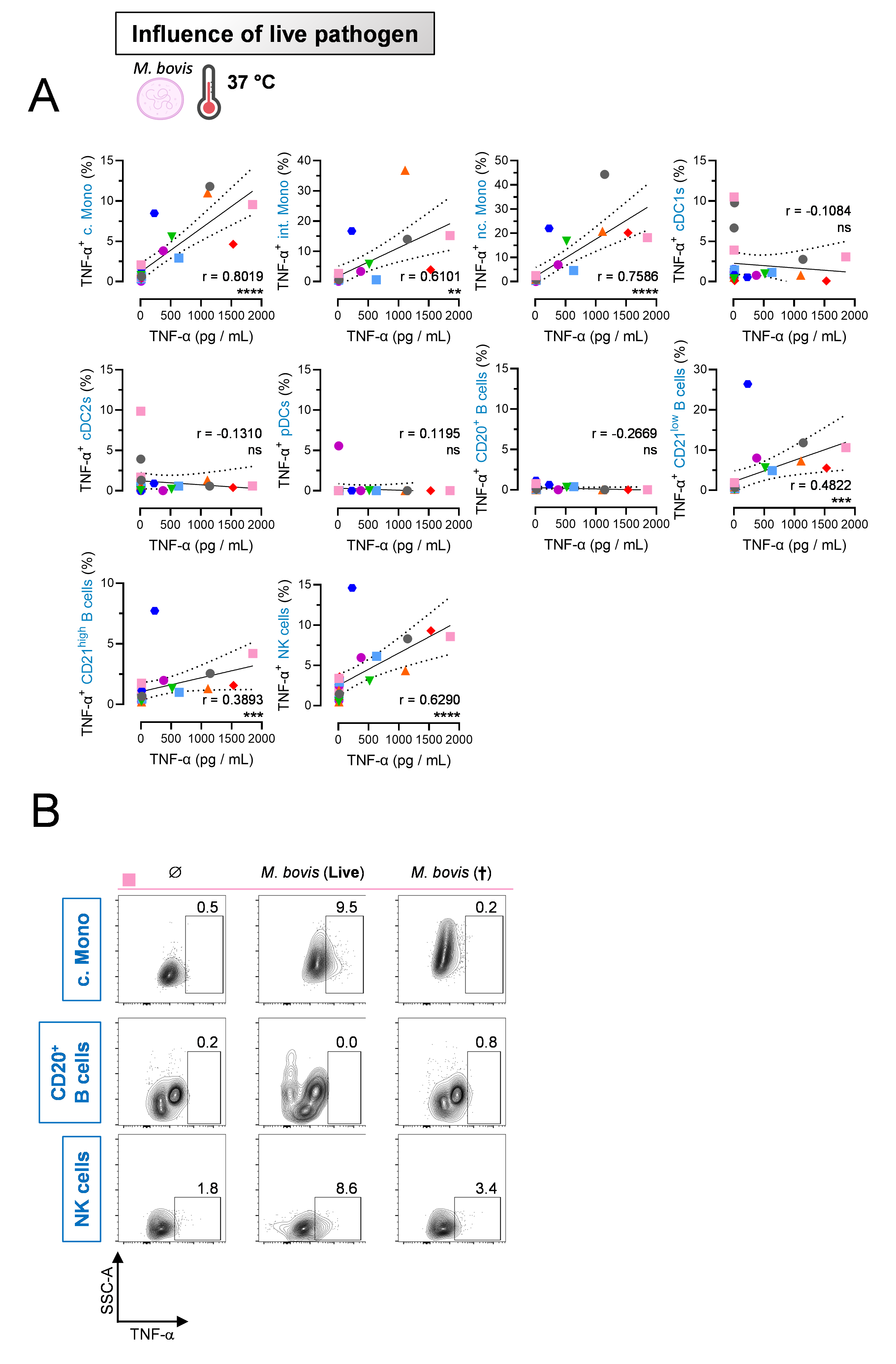

Supplement: Supplementary file 8 — Additional file 8: TNF-α production in response to Mycoplasmopsis bovis is a concerted action of monocytes, B cells and NK cells. A Correlation coefficient (r) obtained with percentage of TNF-α+ cells for different immune cell subsets (measured by FCM) calculated as a function of TNF-α concentration (pg/mL) in culture supernatant (measured by multiplex immunoassay). Each symbol represents an individual animal. Stars indicate significance levels. ***, p < 0.001; ****, p < 0.0001. B FCM contour plots from a representative animal, gated on TNF-α-producing cells (classical monocytes and NK cells, where a correlation was found) and non-producing cells (CD20+ B cells, where no correlation was found). Numbers indicate the percentage of TNF-α+ cells for this specific animal. [file 13567_2024_1272_MOESM8_ESM.tif]

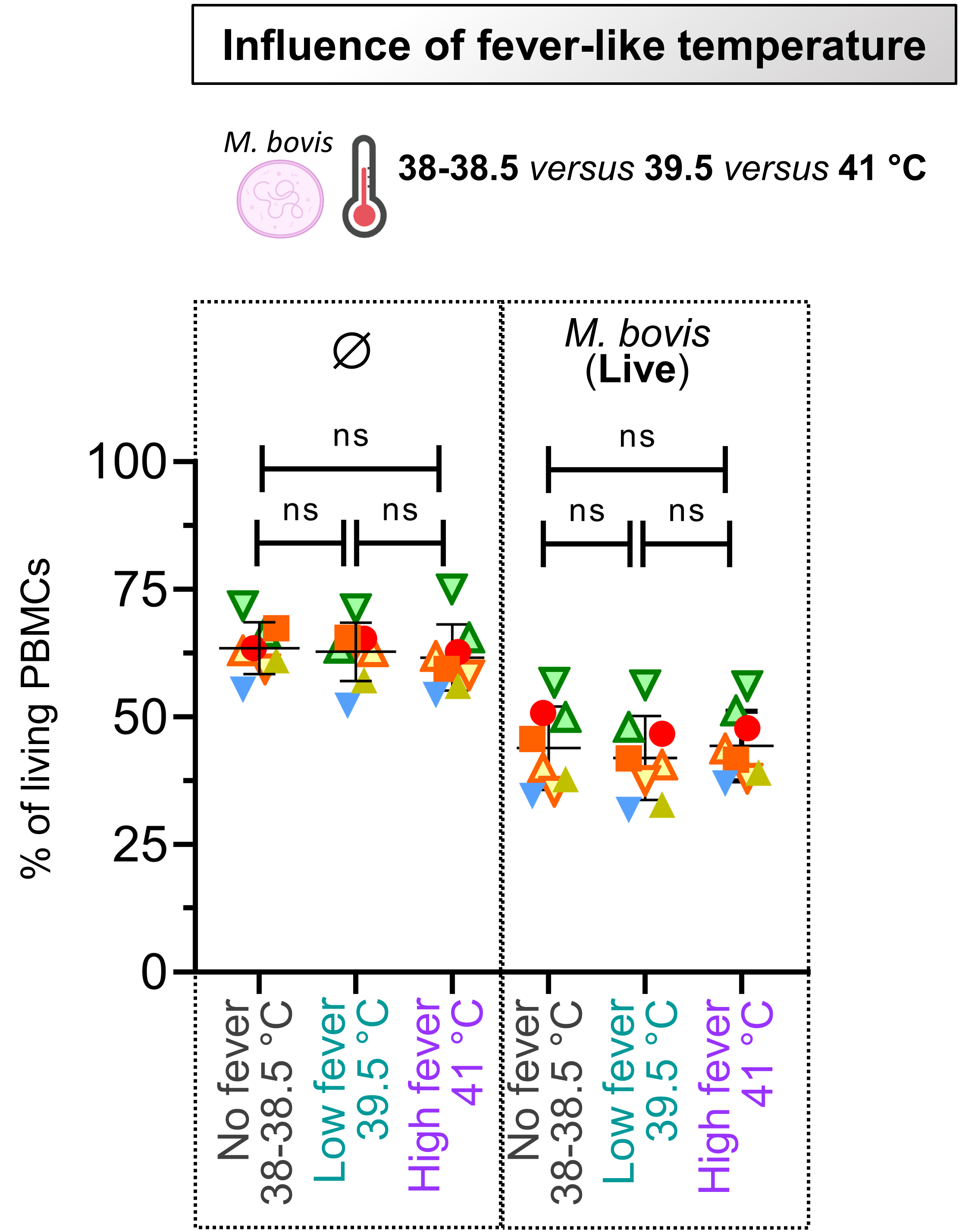

Supplement: Supplementary file 9 — Additional file 9: Impact of the rise of temperature on viable cell frequencies. A primary gate (P1) was set on FSC-A versus SSC-A, then, the percentage of living PBMCs (negative for Live/Dead marker) was quantified by FCM. Cells were let either unstimulated (∅), either stimulated with M. bovis at 3 different incubation temperatures: 38 °C (“No fever”), 39.5 °C (“Low fever”) and 41 °C (“High fever”). Each symbol represents an individual animal and is the average of three independent measurements (“Antigen presenting cells”, “T cells”, and “B cells, NK cells” panels). ns: not significant. [file 13567_2024_1272_MOESM9_ESM.tif]
